# Transfusion-transmitted infections: risks and mitigation strategies for Oropouche virus and other emerging arboviruses in Latin America and the Caribbean

**DOI:** 10.1016/j.lana.2025.101089

**Published:** 2025-05-05

**Authors:** Andres Moreira-Soto, Ignacio Postigo-Hidalgo, Ximena Tabares, Yannik Roell, Carlo Fischer, Eduardo Gotuzzo, Thomas Jaenisch, José Eduardo Levi, Yaniv Lustig, Jan Felix Drexler

**Affiliations:** a Institute of Virology, Charité-Universitätsmedizin Berlin, Corporate Member of Freie Universität Berlin and Humboldt Universität zu Berlin, Berlin, Germany; b Tropical Disease Research Program, School of Veterinary Medicine, Universidad Nacional, Costa Rica, Costa Rica; c Center for Global Health, University of Colorado, Denver, USA; d Instituto de Medicina Tropical Alexander von Humboldt, Universidad Peruana Cayetano Heredia, Lima, Peru; e Heidelberger Institut für Global Health, Universitätsklinikum Heidelberg, Heidelberg, Germany; f LIM-52, Instituto de Medicina Tropical, Faculdade de Medicina da Universidade de São Paulo, São Paulo, Brazil; g Sackler School of Medicine, Tel-Aviv University, Tel-Aviv, Israel; h Central Virology Laboratory, Public Health Services, Ministry of Health, Sheba Medical Center, Ramat Gan, Israel; i German Centre for Infection Research (DZIF), Associated Partner Site Berlin, Germany

**Keywords:** Blood donation, Nucleic acid detection, Vectors, Transfusion-transmitted infections, Cost-effectiveness

## Abstract

Arboviruses impose a major public health burden in Latin America and the Caribbean due to widespread and potentially severe infections causing microcephaly and long-lasting arthralgia. Beyond canonical vector-borne transmission, the magnitude and risk factors for transfusion-transmitted infections (TTIs) are unclear. In this narrative report, we use analyses of virological data such as infection symptomatology, viremic periods, and viral loads, to argue that dengue, Oropouche, Zika, yellow fever, and Chikungunya viruses pose an under-investigated risk of TTIs. An analysis of socioeconomic data showed that blood donation rates in Latin America and the Caribbean correlated with gross domestic product (r = 0.53, *p* = 0.0021) and health expenditure (r = 0.5, *p* = 0.0045), highlighting that resource limitations impact blood screening. Risk maps based on vector occurrence and ecological variables showed that Central America and Northwest coastal Brazil are high-risk zones, making surveillance, vector control, vaccination, and cost-effective blood screening crucial for mitigating TTIs, including Zika and potentially Oropouche viruses in pregnant women.

## Introduction

Latin America and the Caribbean are hotspots of arthropod-borne viruses (arboviruses) due to their diverse tropical ecosystems, each with unique environmental conditions supporting a high variety of vectors and hosts.^1^ The primary arbovirus vectors in Latin America, *Aedes aegypti* and *Aedes albopictus*, are responsible for transmitting dengue (DENV), Zika (ZIKV), and Chikungunya (CHIKV) viruses.^2^ Vector geographic expansion led to the re-emergence of DENV in previously controlled areas, such as Argentina at the end of the 20th century^3^ and aided in the CHIKV and ZIKV epidemics,^1^ which affected millions of people since 2013. *Haemagogus* mosquitoes and *Culicoides* midges are major vectors of Mayaro (MAYV) and Oropouche (OROV) viruses, respectively, and are implicated in recurring infections^4^ in Latin America. Moreover, the recent increase in molecular detection of OROV cases, reaching more than 20 thousand cases in Bolivia, Brazil, Colombia, Cuba, Ecuador, Guyana, Panama and Peru in 2024, along with the molecular detection in stillbirth cases and OROV-specific antibodies suggesting acute infections in six complicated pregnancies in several Brazilian states, is concerning due to its similarity to the Zika outbreak during 2014–15.^5^

Arbovirus infections can occur through vertical transmission, sexual contact -such as with ZIKV^6^-, organ transplantation such as with West Nile virus (WNV),^7^ or the transfusion of infected blood or blood components. Transfusion-transmitted infections (TTIs) encompass a variety of pathogens, including viruses, bacteria, and parasites, and have been documented for DENV,^8^ ZIKV,^9^ and WNV,^10^ as well as for yellow fever virus (YFV) from donors recently vaccinated with the live-attenuated yellow fever vaccine.^11^ Arboviral TTIs may significantly burden healthcare systems due to the high incidence of these often asymptomatic infections,^12^ which are compatible with apparently healthy donors. In 2020, more than 8.2 million units of blood were collected in Latin America, with 1,555,672 patients transfused in six countries alone.^13^ While many Latin American countries screen for blood-borne parasites such as *Trypanosoma cruzi*, bacteria such as *Treponema pallidum*, and viruses such as hepatitis B (HBV) and C (HCV) viruses and for human immunodeficiency virus (HIV),^14^ blood screening for arboviruses is not mandatory. This renders the incidence and risk of arboviral TTI largely unknown. Furthermore, challenges such as test sensitivity, detection window periods, and resource constraints hinder effectiveness, especially in resource-limited settings. However, the potential for debilitating sequelae documented for CHIKV and other arbovirus infections calls for comprehensive strategies to manage arboviral TTIs, particularly in high-risk regions such as Latin America and the Caribbean.^14^

### Search strategy, selection criteria and risk factors of arboviral TTIs

References in English, Portuguese, and Spanish were identified through PubMed, Web of Science and Google Scholar searches with the terms: “transfusion-transmitted infections” OR “transfusion-transmitted infection” AND “arboviruses” OR “arbovirus” OR “arthropod-borne viruses” AND i) “Latin America”, ii) “regulations”; “blood screening” AND “cost-effectiveness”; “blood donations” AND “Latin America”.

To assess the risk of TTIs linked to each of the viruses included in this work: WNV, DENV, ZIKV, YFV, CHIKV, OROV, and MAYV, we analyzed potential risk factors which included: the potential for severe disease, the mean rate of symptomatic disease, the mean viral load in cases, and the mean viremic period. For this, the virus name (example “ZIKV” OR “ZIKA” OR “ZIKA VIRUS”) was searched in combination with the following terms: “symptoms”, “viremia”, “transfusion-transmission risk estimates”, “seroprevalence”, “severe disease” and “viral loads”. We additionally explored further references as appropriate.

### Estimation of risk maps of arboviral disease transmission

Risk maps were generated by combining the corresponding vector distribution map for each vector (Supplementary Figure S1) and six supporting maps detailing elevation, mean annual temperature, precipitation, the presence of vectors and nonhuman primates, human population density and median daily income (Supplementary Figure S2). The characteristics and sources of the vector distribution maps and supporting maps are described in Supplementary Table S1. Areas lacking daily income data (Venezuela and the Colombian Amazon region) were assigned a mean value of 1 in the reclassification. The distribution map of each vector and the supporting maps were subsequently summed (Supplementary Tables S2 and S3). The risk scale for the maps ranged from 0 to 14, except for the *Culex* spp. map (risk scale 0–12), which does not incorporate data from nonhuman primates, as there is only inconclusive evidence of WNV antibodies in nonhuman primates from Latin America.^15^ The lack of data on vector distribution, as in the case of *Haemagogus* spp. and *Sabethes* spp. (Supplementary Figure S1), limits the representation of YFV risk. Similarly, the lack of data regarding the environmental preferences of *Sabethes* spp. prevented us from producing a risk map for this vector. All maps were created with ArcGIS Pro 2.8. A detailed description of the methodology used can be found in the Supplementary Material.

## Part I: limitations of arbovirus screening in Latin America

### Poor surveillance for non-routinely screened pathogens and suboptimal monitoring

The lack of surveillance of non-routinely screened pathogens in blood may entail underestimations of the number of TTIs caused by arboviruses.^16^ In Latin America, most countries have blood transfusion screening programs (99.8% donations screened)^17^ against serological markers of HIV, HBV surface antigen, HCV, *T. pallidum* and *T. cruzi*. Some countries, such as Brazil, Ecuador, Panama and some Argentinian provinces, introduced the mandatory use of molecular tests for the detection of viral RNA of HIV and RNA/DNA of HBV and HCV.^18^ This approach, in combination with improved donor selection criteria and antibody testing, has proven to be effective in the USA, where blood safety regulations resulted in a 10,000-fold lower risk of contracting TTIs caused by HIV, HBV or HCV in 2010 compared to the 1980s.^19^ However, decisions on screening methods are made at the institutional level based on local epidemiological needs,^20^ as regulations often lack specific guidelines. For example, in the US, mandatory screening of blood donations for WNV is required,^21^ but the regulation does not specify the method. Therefore, institutions must assess the health benefits versus costs of screening methodologies individually.^22^

Accurate serodiagnosis of arboviruses is limited by the cross-reactivity of antibodies with conserved viral epitopes of cocirculating viruses and by the lack of locally validated serological tests.^23^ Screening programs performed in many Latin American countries include serological and molecular screening, with 73% of programs relying on serology to assess TTIs.^17^ Adding nucleic acid testing (NAT) to the serological screening of blood donations enables early detection of pathogens during the pre-seroconversion phase, decreasing the residual risk of TTIs during this period.^24^^,^^25^ While NAT can be conducted *in-house* with reliable controls, its implementation is hindered in resource-limited settings by the scarcity of automated platforms.^26^ Nonetheless, NATs also have methodological limitations, for instance, minipool testing (MP-NAT) shows low sensitivity, increasing the chance of overlooking donors with low levels of viremia.^27^ For example, WNV screening in the US first adopted MP-NAT but later changed to individual donations (ID-NAT) since one-third of RNA-positive donations were missed due to low viral loads.^28^ This underscores the need to characterize the viraemia levels of emerging arboviruses to improve testing protocols and assess transmission risks. In many Latin American countries, NAT for arboviruses is not routinely performed due to the lack of validated assays and its lack of data assesing blood safety.

### Low-income countries struggle to meet transfusion needs due to insufficient blood donations

**Fig. 1:**
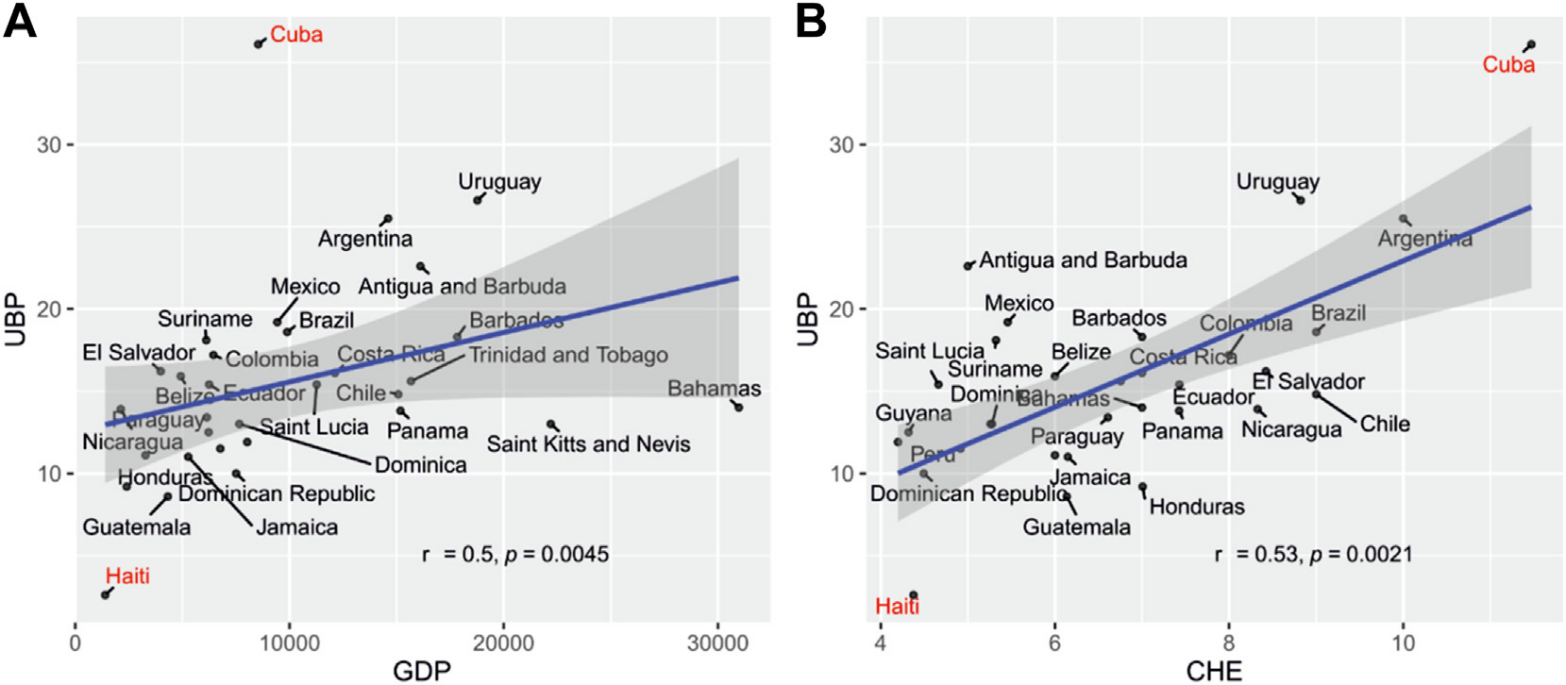
**Economic aspects of blood donations. A**. Spearman correlation (r) and significance value (*p*) between the blood donation rate of Latin American countries, calculated in units of blood per thousand population (UBP), and gross domestic product (GDP in millions of US$). **B**. Spearman correlation (r) and significance value (*p*) between the blood donation rate (UBP) and current health expenditure (CHE as a percentage of GDP). The gray shadow indicates the 95% confidence interval. Data on the blood donation rate, CHE and GDP for 2017 were retrieved from the Pan American Health Organization.^17^

Blood donations in many Latin American countries are often insufficient to sustain the demand for transfusions.^14^ The COVID-19 pandemic exacerbated this trend, leading to 20% fewer units of blood in Latin America than in 2017.^13^ Differences in blood donation rates among Latin American countries are related to the country's gross domestic product (GDP), a trend observed worldwide,^29^ which was also observed in our study (r = 0.53, *p* = 0.0021) (Fig. 1A). Notably, Haiti, the country with the lowest GDP in the region, had a blood donation rate of 2.6 units of blood per thousand (UBP)^17^ in 2017, the lowest among the analyzed countries. In addition, a country's health expenditure (CHE) seems to be linked to increasing its blood donation rate (r = 0.5, *p* = 0.0045) (Fig. 1B). In concordance, Cuba, the country with the highest health expenditure in Latin America and the Caribbean (11% of its GDP), despite having a low GDP, had the highest blood donation rate in the region (36.1 UBP in 2017).^17^ However, these are extreme examples that should be taken with caution.

Regarding the source of blood products, voluntary donors are considered safer than replacement donors (on demand family/community-supplied transfusions).^17^ In 2014, Latin American countries approved the “Action Plan for Universal Access to Safe Blood”, emphasizing voluntary donations, organizing blood services, and ensuring quality and safety.^13^ However, in 2020, only 48% of donations were voluntary,^13^ while the rest were replacement donations.^17^ In low-income settings, most transfusions occur during emergencies such as childbirth hemorrhage and malaria-related anemia in children, necessitating immediate blood availability.^30^ This poses a safety risk for pregnant women since in emergency situations, they might receive donated blood that might cause fetal or newborn developmental complications. Additionally, recruiting and retaining unpaid volunteers significantly increases costs, up to four times more than replacement donations.^30^ Thus, a mix of replacement and volunteer donors is often more practical and cost-effective.^30^ Targeting screening efforts to voluntary blood donations would provide a pool of safe blood products available for emergencies, balancing sufficiency with safety (Table 1).

**Table 1:** Problems and solutions in the management of arboviral transfusion-transmitted infections.

General category	Specific category	Problem	Recommendation	Solution
Diagnostics	Screening standards	Inadequate blood safety standards and poor molecular screening practices underestimate TTI from arboviruses.	Enhance transfusion safety standards	Prioritize threats and implement molecular screening based on evidence and regulations.
	Serological tests	Screening relies on imprecise serological tests due to antibody cross-reactivity and lack of local validation.	Adopt more precise testing methods	Shift to molecular screening for early pathogen detection and reduced TTI risk.
	TTIs risk values	Challenges exist in calculating TTI risk values for arboviruses, with estimates mainly valid for endemic outbreak areas.	Standardize TTI risk scoring	Create an overall virus TTI risk score and develop algorithms for safer blood testing.
	Population representation	Blood donor-based serosurveillance has limited representativeness, leading to biased risk estimates.	Widen serosurveillance coverage	Include various population groups and use surveillance measures and risk mapping for control strategies.
Logistics	Donation quality	The minority of blood donations come from voluntary donors.	Foster voluntary blood donations	Encourage voluntary donations, improve blood services, implement safety standards, and focus screening on voluntary donations.
	Donation rates	Insufficient and unevenly distributed blood donations, especially in low-income countries.	Boost health sector investment	Increase health spending to raise blood donation rates.
	Screening programs	High costs lead countries to discontinue arbovirus blood screening programs, despite long-term benefits.	Weigh long-term benefits against costs	Sustain viral surveillance strategies and NAT screening for arboviruses, especially during outbreaks.
Regulatory	Regulatory guidelines	Lack of specific guidelines for screening methods and limited access to effective NAT.	Establish comprehensive blood safety regulations	Integrate serological and NAT into screening processes, develop local multiplexed assays, and explore treatment alternatives.
	Outbreak screening	Difficulty identifying virus transmission during outbreaks and lack of seasonal screening strategy.	Develop flexible outbreak management strategies	Adopt blood screening methods as per epidemiology. Apply a One Health approach, restrict asymptomatic donors temporarily, and use predonation questionnaires.

NAT: nucleic acid testing, TTI: transfusion-transmitted infection.

### Suitable transfusion transmission risk estimates are missing

TTI risk estimates are crucial for controlling and maintaining a safe blood supply. However, Latin America and the Caribbean lack global arboviral TTI risk estimates. Current estimates are mainly relevant for endemic areas with reported outbreaks (Table 2). The risk of arbovirus transmission varies among individuals affected by transfusion conditions and health status. For DENV-TTIs, not every RNA-positive donation is infectious,^76^ indicating that factors such as viral amount, stability, transfused blood volume, and recipient immune status play roles in transmission and disease. Moreover, TTI risk involves intricate factors, including viremia duration, symptom presence, disease incidence variability, and viral load differences,^2^ complicating an accurate assessment. The high risk of DENV-TTIs is related to dengue endemicity and high prevalence across Latin America. In 2023–2024, the American region experienced the largest dengue epidemic on record since 1980, with over 12.6 million reported cases and nearly 21,000 severe cases.^79^

**Table 2:** Overview of arbovirus characteristics, prevalence, and transfusion transmission risk factors in Latin America and the Caribbean.

Virus	Proportion [%] of severe disease^d^	Main vector in Latin America	Mean rate and range [%] of symptomatic disease	Risk of TTIs per 10k persons	Risk of viremic donations per 10k persons	Seroprevalence in blood donors (IgG, %)	Viral load in blood donors [range, copies/mL]	Viral load in cases [range, copies/mL]	Viremic period [range, days]
Oropouche	6.1 of 165 hospitalized patients with suspected CNS infection^31^	*Culicoides paraensis*^(^^4^^)^	63^(^^32^^)^	ND	ND	ND	ND	[5.42 × 10^4^–1.65 × 10^8^]^s(^^33^^)^	[1–5]^(^^34^^)^
West Nile	<1.0^35^	*Culex* spp.^(^^36^^)^	20^(^^37^^)^	1.8–2.7 New York-outbreak^(^^38^^)^	2 New York^(^^38^^)^ 0.3–1.49 US^(^^37^^,^^39^^)^	8.2 Tunisia^(^^40^^)^ 10.4 Qatar^(^^41^^)^ 4.2–19.7 US^42^	[10^2^–5 × 10^3^]^p(^^43^^)^ [10^2^–5 × 10^4^]^wb(^^43^^)^	[1–4.72 × 10^5^]^wb(^^44^^)^ [10–10^3^]^p(^^44^^)^	[2–13.2]^(^^38^^)^
Dengue	0.08–7.4, Americas, 2022^45^	*Aedes* spp.^(^^46^^)^	30 [13–47]^(^^47^^)^	0.5 Australia-outbreak^(^^48^^)^ 1.6–6 Singapore-endemic^(^^49^^)^	7 Puerto Rico^(^^50^^)^	24^(^^51^^),^^a^ 1–20 Puerto Rico^(^^20^^)^	[10^5^–10^9^]^p(^^52^^)^	[10^3^–10^8^]^s(^^46^^)^	[4.4–13.9]^(^^53^^)^
Zika	4.6-6 incidence of birth defects^54^	*Aedes* spp.^(^^55^^)^	38.2 [12.9–67]^(^^12^^),^^a^	ND	89 Puerto Rico during epidemic in 2016^(^^55^^)^ 0.02 US^(^^56^^)^	5.1^(^^51^^),^^a^ 1.61^(^^57^^),^^a^	[2.5 × 10^3^–8.1 × 10^6^]^p(^^58^^)^	[10^2^–2 × 10^6^]^s(^^59^^)^	[4–6]^(^^60^^)^ [16–48]^c^^,(^^61^^)^
Yellow fever	12.0, modeled for tropical areas^62^	*Haemagogus* spp. *Sabethes* spp.^(63^^)^	45^(^^62^^)^	ND	ND	0.6 Turkey^(^^64^^)^	ND	[10^2^–10^7^]^(^^63^^),^^a^	[3–10]^(^^20^^)^
Chikungunya	0.6–13.0 Hospitalization rates due to arthralgia^65^	*Aedes* spp.^(^^66^^)^	72 [65–80]^(^^66^^)^	4.1 Thailand-outbreak^(^^67^^)^	3.8–5.2 Thailand^(^^67^^)^ 13.2 Reunion Island^(^^68^^)^	21.6^(^^51^^),^^a^ 37.2 Reunion Island^(^^68^^)^	[2.9 × 10^5^–9.1 × 10^7^]^p(^^69^^)^	[1.7 × 10^5^–9 × 10^9^]^p(^^70^^)^	[1.5–7.5]^(^^68^^)^
Mayaro	20 cases with long-lasting arthralgia^71^	*Haemagogus* spp.^(^^4^^)^	70 [50–90]^(^^72^^)^	ND	ND	0.19 Brazil^(^^73^^)^	ND	1.24 × 10^7s(^^74^^),^^b^	[2–5]^(^^75^^)^

TTIs: Transfusion-transmitted infections. ND: No data found. Components used for viral load diagnosis quantification: wb: whole blood; p: plasma; s: serum. Ranges are indicated in square brackets. The reference studies are shown in parentheses.

a Pooled values from different studies referenced in the Supplementary Methods.

b Single case value.

c Value obtained from whole blood samples.

d Congenital infection; acute neurological disease; neurological disease; shock; hemorrhage, Guillain-Barre, hepatitis; arthralgia; CNS: central nervous system.

Other emerging arboviruses appear to have a high TTI risk, namely, OROV, ZIKV, WNV, YFV and CHIKV. YFV TTIs are rare, but there is a recent report of a 17D YFV-vaccinated individual who infected three transfused individuals due to a lack of YFV screening in the US.^11^ WNV accounts for the largest number of TTIs worldwide. Nevertheless, the risk of WNV infection in Latin America remains unclear, as WNV endemicity or enzootic circulation has not been fully confirmed.^80^ Furthermore, only a few cases of human WNV infections have been reported in Latin America, which may be related to unreported asymptomatic infections or to cross-protection provided by previous infections with other flaviviruses.^80^ WNV-TTIs and DENV-TTIs pose risks of complications for transfusion recipients, such as hemorrhagic fever or encephalitis for immunosuppressed patients.^8^^,^^10^

ZIKV remains a concern due to its potential to cause fetal malformations or pregnancy losses.^61^ Despite a decline in the regional incidence of ZIKV post-2016, stable transmission has occurred in countries such as Belize, Brazil, and Paraguay.^81^ Furthermore, ZIKV-TTI risk may fluctuate based on the average duration of viremia, which ranges from several days to more than a month,^82^ depending on whether the viral load is quantified in serum or whole blood.

The OROV is an emerging concern in Latin America. In early 2024, the Brazil IHR National Focal Point (NFP) reported a cluster of OROV cases in the states of Amazonas, Acre and Roraima that were being investigated.^83^ As of November 2024, over 10,000 confirmed cases of OROV were reported from Latin America, and recent near continent-wide antibody data suggest that OROV infections may always have been under-detected in numbers and geographic spread.^84^ Additionally, two deaths^85^ (https://www.paho.org/en/epidemiological-alerts-and-updates) in adults, 2 cases of fetal death, confirmed by OROV-specific molecular detection methods, and six cases of complicated pregnancies with microcephaly and also positive for acute serological markers of infection potentially attributed to OROV were reported from Brazil.^5^

CHIKV and MAYV can have a high risk of TTIs. However, these viruses are of particular concern because their proportion of asymptomatic infections is poorly understood.^4^ The reported incidence of symptomatic CHIKV infections, for example, ranges from 20% to 72% in Latin America.^86^^,^^87^ Due to symptom overlap with DENV and MAYV (e.g., high fever, arthralgia, weakness), CHIKV infections are often misdiagnosed, underestimating true symptomatic infection rates.^86^ In summary, assessing the potential risk of TTIs, and developing regional or local data-driven algorithms are needed for safer blood testing. These algorithms consider factors such as the local prevalence of diseases, transmission seasonality, and demographics, enabling improved and potentially cost-effective blood donor screening (Table 1).

## Part II: cost-effectiveness of arbovirus blood screening

### Arbovirus blood screening efforts are tied to recipients’ disease risk

Regardless of their costs, implementing and supporting viral surveillance strategies often depends on assessing their effectiveness, considering the potential risk of transmission and severe disease in recipients. In the US, WNV screening of blood donations has been mandatory since 2003,^21^ as 56.8% of reported TTIs have been associated with WNV.^16^^,^^88^ However, the cost of this procedure was estimated to be four times greater than what is accepted as a threshold for medical procedures in the US.^22^ Despite the economic impact of DENV, calculated as US$46.4 million between 2002 and 2010 in Puerto Rico, which is five times the cost of surveillance and vector control,^89^ blood product screening for DENV was only introduced in a handful of Latin American countries, including Puerto Rico.^25^ The limited screening was probably due to the lack of DENV-TTI-related deaths^16^ and an estimated low risk of developing severe dengue after transfusion.^77^

Similarly, for the Latin American region, the economic cost of neurological disease caused by ZIKV has been estimated at US$2.3 billion per year,^90^ challenging Latin American social and health systems. The 2015 Zika epidemic in Latin America prompted a ZIKV-specific NAT to be implemented in 2016 in the US, i.e., Puerto Rico. However, as with DENV-TTI, recipients of infected units did not develop severe symptoms associated with ZIKV infection.^9^ In Puerto Rico, between 2017 and 2018, only a few cases of ZIKV infection were detected by donation^25^; for example, with DENV-TTI, the associated costs were 10 times greater than the threshold commonly accepted for clinical procedures.^91^ Guidance recommending ZIKV testing of donors was withdrawn in 2021 in the US due to its low prevalence.^92^ Finally, in the European Union, the deferral of blood donations from WNV-affected areas and potentially exposed persons has been enforced since 2004, unless a negative individual NAT result is presented.^93^ As a result, some countries implemented NAT screening during outbreaks to compensate for the reduction in blood donations available at the national level, as was the case for Italy during the WNV outbreak in 2008.^94^

### Incidence-based arbovirus blood screening strategies

The search for alternatives to optimize the cost‒benefit of blood screening has led to the adoption of different approaches according to specific epidemiological conditions, such as high or low incidence areas and outbreak conditions. Seasonal NAT screening of individual samples in high-incidence areas has been estimated to be cost-effective for detecting WNV and ZIKV, particularly when restricted to blood donations intended for at-risk populations such as immunocompromised patients and pregnant women.^22^^,^^78^ As an affordable and cost-effective alternative for WNV blood screening in Italy, a *One Health* approach has been adopted since 2013, which, upon confirmation of WNV in mosquitoes, birds or horses in a given province, triggers routine ID-NAT of blood donations from that province until the end of the annual transmission season, avoiding additional testing during the following transmission season.^95^ In low-incidence areas and non-endemic regions, deferring potential donors to visiting outbreak settings impacts donor loss. Strategies to guarantee transfusion safety without jeopardizing blood availability include (i) temporary restriction of asymptomatic individuals to donate for a limited period of time and (ii) post-donation symptom follow-up for 14 days and eventual quarantine of blood products.^58^ In principle, all these criteria could apply for the current increase in OROV cases in Latin America. In Colombia, blood banks located in non-endemic areas carry out a temporary 4-week deferral for visitors from risk areas or with circulation of DENV, YFV, CHIKV and/or ZIKV, as well as for donors with symptoms or previous infection by ZIKV and CHIKV, following symptom clearance.^96^

Finally, during outbreaks such as the 2024 increase in OROV cases in Latin America, identifying TTIs can be challenging, as vectors can independently infect donors and recipients. Thus, seasonal screening may help to maintain transfusion safety,^25^ but this strategy has not been harmonized, and no guidance is available on when to start donor screening based on the transmission intensity or force of infection, understood as the number of infections acquired per person per year.

### Cost challenges of nucleic acid tests for routine blood screening in Latin America

The routine use of NATs in Latin America is hampered by their high costs and limited accessibility. In 2017, NAT costs were estimated to be between US$10 and 14 per blood donation for MP-NAT and between US$14 and 24 for ID-NAT.^97^ The implementation of NAT screening for HIV, HCV and HBV, for example, is considered cost-effective when it is equivalent to US$ 1/donor relative to other health interventions.^98^ However, in 2011, related NAT costs in Ecuador were US$ 20/donor, with tests performed on six donor minipools, while in Colombia, costs were approximately US$ 15/donor.^24^^,^^99^

The cost-effectiveness of NAT is also limited by the fact that 50% of Latin American countries manage blood collection and processing in small blood banks.^13^ These banks process less than 5 thousand units of blood per bank yearly, and their main source of supply is replacement donations.^13^ Considering the high costs of reagents, supply costs per donor in small banks are high and can be up to three times the supply costs of larger banks that process more than 25 thousand units of blood per year.^100^ However, small banks favor population access to transfusion services outside major urban centers. The local development of multiplexed assays and their commercialisation in the region would reduce the costs of screening, making them cost effective for small blood banks (Table 1).

### Cost-effective alternatives to blood screening for the mitigation of TTI

There are alternatives to blood screening to mitigate TTI risks. For example, the use of standard pre-questionnaires to avoid potential symptomatic donors has proven to be cost-effective, as in the case of WNV.^22^ A fuller understanding of the proportion of symptomatic infections caused by DENV, MAYV, OROV, CHIKV and YFV would also allow this strategy to be applied in high-incidence settings, as these viruses are likely to cause symptoms in more than 45% of infections (Table 2).

Vector control interventions present another cost-effective option, with costs ranging from US$5 to US$40 per household,^101^^,^^102^ given the increased control effectiveness in the general population.^14^ Strategies such as insecticides and water container management can effectively increase mosquito-pupal mortality, reducing indoor abundance to approximately 50%.^103^ However, long-lasting effects require broad, intensive and sustained application of diverse vector control techniques.^104^ With dengue, vector control can temporarily curb transmission, but it may also diminish local immunity, heightening the risk of future outbreaks.^105^ Similarly, vector population density metrics of *A. aegypti* were not correlated spatially with the locations of Chikungunya fever recurrence in Ceará and Tocantins, Brazil.^106^ Combining vaccination with vector control, as proposed for other arboviral diseases such as yellow fever, might be more effective.^107^

Pathogen reduction technology (PRT) offers an economical alternative to donor testing and deferrals, enhancing blood supply.^108^ PRT employs photoactive compounds to neutralize pathogens while largely preserving blood components, an advantage for pathogens without available blood tests. For example, amotosalen-UVA-treated platelets proactively mitigate ZIKV-TTI risks^58^ and reduce those from WNV, DENV, and CHIKV.^108^ However, PRT is only approved for platelets and plasma, increasing the cost to US$60–110 per donation.^97^ Whole-blood PRT could halve this expense, boosting cost-effectiveness through donor retention and ensuring safer blood derivatives such as platelet concentrates or fresh-frozen plasma.^97^

Detecting arboviruses such as ZIKV and WNV is more efficient in whole blood. Given that red blood cells account for 47% of documented TTI cases as of 2021 and 55% of Latin American transfusions as of 2020, exploring and approving treatment alternatives for whole blood and/or red blood cells is crucial to enable a cost-effective treatment to mitigate the risk of TTIs (Table 1).

Finally, broadly reactive flavivirus NAT would be advantageous for covering DENV and WNV, which account for the majority of documented TTIs, as well as potentially new threats such as ZIKV, Saint Louis Encephalitis Virus and YFV, and potentially CHIKV, DENV and YFV live vaccines.^109^ However, available assays relying on conserved genomic regions to design a generic test relying on a few oligonucleotides only are likely not sufficiently sensitive for blood product screening.^110^ Therefore, new techniques that can combine several highly sensitive assays multiplexed on high-throughput platforms are essential^110^ and should be urgently developed. Ideally, OROV- and CHIKV-/MAYV-specific tests in addition to the flaviviruses ZIKV, DENV and WNV could be included in such approaches.

## Part III: preparedness for new viruses and outbreaks

### Surveillance data are essential but limited in assessing arboviral transfusion-transmitted infections

Surveillance data on TTIs are crucial for understanding disease prevalence and guiding decisions on prevention and outbreak preparedness. Blood donor-based serosurveillance^111^ offers a cost-effective method for determining arbovirus prevalence, aids in epidemiological forecasting of outbreaks and has been previously employed for COVID-19 prevalence studies.^112^ In fact, a correlation exists between donor seroprevalence growth and incidence in the general population.^111^^,^^112^ On the other hand, using donors as a surveillance tool can present several challenges: (i) risk estimation accuracy, as donors are typically healthier due to recruitment criteria, seroprevalence might be underestimated^113^ (Table 2); (ii) the fact that donors often do not reside in donation areas could introduce bias; (iii) donor demographics often exclude groups such as children and elderly individuals, limiting representation; (iv) time lags in serology, since IgG antibodies are usually not present before two to three weeks after acute infection,^23^ and (v) exposure to different yet antigenically related viruses can affect limits the sensitivity and specificity of serological tests in endemic areas, underscoring the need for exhaustive validation.^23^

### Leveraging risk maps for effective vector control and arbovirus outbreak preparedness

Estimating potential outbreaks requires multivariate modeling that accounts for ecological changes and their impact on the virus vector and host environment. Changes in ecological conditions (temperature, rainfall), land use (urbanization, mining), and the mobility of goods and people have been proven to affect virus vector/host abundance and thus the risk of TTIs.^114^ For instance, changes in temperature are expected to affect the distribution of *A. aegypti* and *A. albopictus*, as they are the main constraints on the survival of both species, while changes in precipitation may mainly affect the abundance of *A. albopictus* due to its preference for laying eggs in non-domestic habitats.^115^ Moreover, inadequate sanitation and domestic water storage activities facilitate the formation of breeding sites.^116^ Little is known about OROV invertebrate vectors and their ecological limitations, but the literature suggests that *Culicoides* spp. including the main OROV vector *C. paraensis* can thrive in urban and rural settings.^117^ Nevertheless, the transmission cycle of OROV in Latin America is poorly understood.^117^ Additionally, the risk of virus introduction or reintroduction persists as infected people or animals migrate from low-incidence areas to densely populated urban centers, entailing enhanced transmission.

**Fig. 2:**
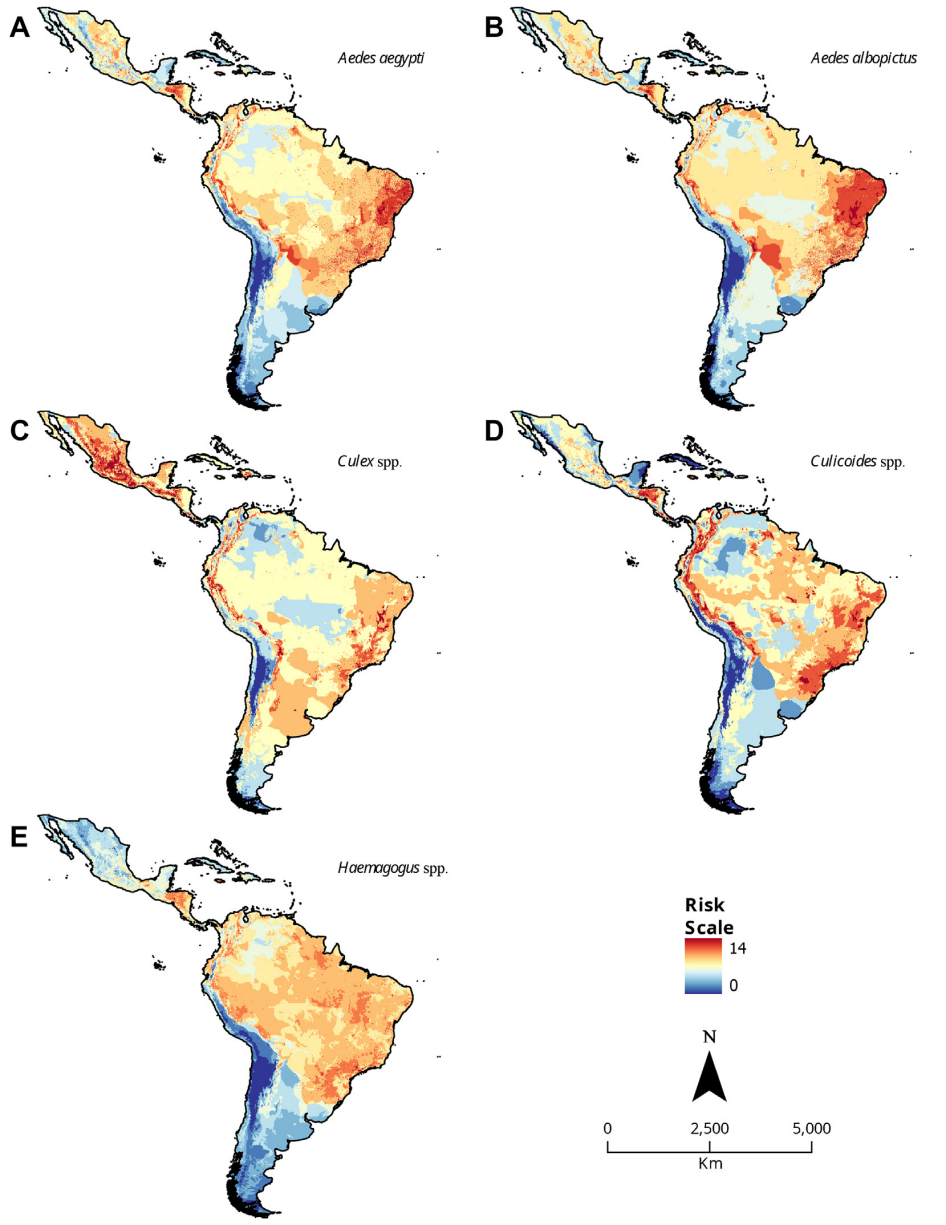
**Risk maps of arboviral disease transmission**. Risk maps indicate hotspots of arbovirus transmission according to the co-occurrence of vector-favorable environmental variables (elevation, mean annual temperature, mean annual precipitation), the presence of vectors and nonhuman primates, human population density and median daily income. **A.** Risk map for *Aedes albopictus*; **B.** Risk map for *Aedes aegypti*; **C.** Risk map for *Culex* spp.; **D.** Risk map for *Culicoides* spp.; **E.** Risk map for *Haemagogus* spp.

Risk mapping is a valuable tool for arbovirus epidemiology because it allows the combination of various factors that contribute to arbovirus transmission, such as the presence of vectors, population density, climate and socioeconomic conditions. Based on the available literature, we developed risk maps for the main vectors in the region (Fig. 2; refer to Supplementary Material for the extended methodology). In Latin America, *Aedes* spp. have high concentrations in tropical lowlands of northeastern, southeastern, and midwestern Brazil (Fig. 2A and B), with rural and peri-urban areas in this region being at high risk for future arboviral disease outbreaks, e.g., DENV. Similarly, Mexico showed a high risk, suggesting the need for special monitoring of potential outbreaks of WNV based on the *Culex* spp. risk map (Fig. 2C). Ecuador and Peru, Colombia, several countries in Central America, Southeast Venezuela and Northeast and Southeast Brazil should be investigated OROV-TTIs, based on the *Culicoides* spp. risk map from this study, and in concordance with a spatial modeling study^84^ (Fig. 2D).

Since *Haemagogus* spp. tolerate lower temperatures than *Aedes* spp*.,* allowing it to establish at higher altitudes, such as in Southern Brazil and Northeastern Argentina or in the foothills of the Eastern Andes in Peru, YFV monitoring and vaccination in these regions are essential (Fig. 2E). It is also worth noting the high vulnerability of low-income settings, such as in El Salvador and Haiti, which offer favorable conditions for the emergence of different vectors. In these regions, the intensification and continuation of vector control, vaccination, and blood product surveillance should be prioritized.

In summary, risk maps offer a comprehensive view of the arbovirus transmission landscape and support decision-making by public health authorities and other stakeholders. However, it is important to acknowledge their limitations, as they are based on a limited set of risk factors that may not fully capture the complexity of virus transmission and mostly rely on presence-only data, which can introduce multiple types of bias (Table 1). Therefore, there is a need to integrate more advanced ecological models ideally combining multiple methods with presence-absence data^84^ and expand data collection on vector populations, alongside TTI monitoring and disease surveillance, particularly in remote areas.

## Conclusions and recommendations

The risk of arboviral TTIs is complex to calculate. The potential for severe disease considers the natural disease course via vector transmission rather than through transfusion, and it is feasible that both probability of infection and disease may be affected by mosquito saliva and skin monocytes^118^ which are absent in TTIs. Although arboviruses can potentially cause severe disease or even death, exemplified by WNV with an 18.9% mortality rate in 74 documented TTIs,^16^ and potentially ZIKV and OROV in vulnerable populations, the lack of information on those emerging arboviruses limits further discussion on their potential to cause severe disease via TTI. In sum, TTIs may not always result in disease and factors underlying the discrepancy between infection and severe disease require urgent investigation for emerging arboviruses with a focus on ZIKV and OROV.

Latin America and Caribbean regions are hotspots for arbovirus emergence. Specifically, DENV screening via blood donations is vital due to the high risk of transfusion-transmission. However, any effective strategy for reducing arboviral transmission risks should align with the region's unique epidemiological and ecological features. A blend of donation screening and sustained vector control is required in high-risk areas identified in our and other studies. Blood screening during outbreak conditions, such as for the ZIKV outbreak in 2015 or the increase in OROV cases since 2023–2024, might warrant cost-effectiveness analyses, noting that screening might be a valuable tool to preemptively identify regionally increased incidence. Screening blood donations can provide important information on whether complications in unborn children are due to the increased geographic spread of OROV infections,^118^ increased overall case numbers, or potentially altered viral phenotypes associated with increased transmissibility in vertebrate or invertebrate hosts.^84^^,^^117^ These initiatives require increased public health funding, a re-evaluation of cost–benefit benchmarks for blood screening, the development of standards for blood surveillance, and institutional coordination. Addressing these factors can help mitigate arboviral risks in Latin America.

## Contributors

XT, IPH, AMS and JFD contributed to the conceptualization, formal analysis and writing of the initial draft. YR contributed to data curation and formal analysis. All authors contributed to draft editing, reference validation, writing– review & editing.

## Editor's note

The Lancet Group takes a neutral position with respect to territorial claims in published maps and institutional affiliations.

## Declaration of interests

The authors have no conflicts of interest to declare.

## Appendix A Supplementary data

Supplementary material

Translated Summary

Translated Summary
