# Supplementary material for: Transfusion-transmitted infections: risks and mitigation strategies for Oropouche virus and other emerging arboviruses in Latin America and the Caribbean
Source: Lancet Reg Health Am. 2025 May 5;46:101089. doi: 10.1016/j.lana.2025.101089 (PMC12127558; doi:10.1016/j.lana.2025.101089)
Supplement: Supplementary material [file mmc1.pdf]

Supplementary material for: "Transfusion-transmitted infections: risks and mitigation strategies for Oropouche virus and other emerging arboviruses in Latin America and the Caribbean"

Authors: Andres Moreira-Soto, Ph.D.[1,2,†]; Ignacio Postigo-Hidalgo, M.Sc.[1,†,] Ximena Tabares, Ph.D.[1,†], Yannik Roell, Ph.D.[3], Carlo Fischer, M.Sc.[1], Eduardo Gotuzzo, MD[4], Thomas Jaenisch, MD[3,5], José Eduardo Levi, Ph.D.[6], Yaniv Lustig, Ph.D.[3,7,8], Jan Felix Drexler, MD[1,9,*].

[1] Institute of Virology, Charité-Universitätsmedizin Berlin, corporate member of Freie Universität Berlin and Humboldt Universität zu Berlin, Berlin, Germany.

[2] Tropical Disease Research Program, School of Veterinary Medicine, Universidad Nacional, Costa Rica, Costa Rica.

[3] Center for Global Health, University of Colorado, Denver, US.

[4] Instituto de Medicina Tropical Alexander von Humboldt, Universidad Peruana Cayetano Heredia, Lima, Peru.

[5] Heidelberger Institut für Global Health, Universitätsklinikum Heidelberg, Heidelberg, Germany

[6] LIM-52, Instituto de Medicina Tropical, Faculdade de Medicina da Universidade de São Paulo, São Paulo, Brazil.

[7] Sackler School of Medicine, Tel-Aviv University, Tel-Aviv, Israel.

[8] Central Virology Laboratory, Public Health Services, Ministry of Health, Sheba Medical Center, Ramat Gan, Israel.

[9] German Centre for Infection Research (DZIF), associated partner site Berlin, Germany

[†] Authors contributed equally

*Correspondence to: Jan Felix Drexler, MD

Institute of Virology, Campus Charité Mitte, Charitéplatz 1, 10098 Berlin, Germany.

Email: felix.drexler@charite.de

Supplementary methodology.

Methodology for the Estimation of Arboviral Disease Transmission Risk Maps

The risk maps presented in this study were generated by integrating vector distribution data with environmental and socioeconomic variables. These maps were created using ArcGIS Pro 2.8 through a series of systematic steps that included data reclassification, raster resizing, and composite raster calculation.

1. **Data Reclassification**:
    - Raster data for environmental variables (e.g., mean annual temperature, precipitation) and socioeconomic variables (e.g., population density, median daily income) were reclassified into discrete categories. This classification was based on the value ranges and assigned scores provided in Supplementary Tables S2 and S3.
        - **Environmental Variables (Table S2)**: Variables such as Mean Annual Precipitation (MAP) and Mean Annual Temperature (MAT) were reclassified into three levels: 0 (none or very low presence), 1 (survival range), and 2 (optimum), according to the known ecological ranges of the vectors studied.

- **Vector and Socioeconomic Variables (Table S3)**: Vector occurrence data were categorized as 0 (non-occurrence) or 1 (occurrence). Socioeconomic variables, including median daily income and population density, were classified based on World Bank poverty indicators and urbanization indices for Latin America, with values assigned as 0, 1, or 2 to represent varying degrees of vulnerability or density.

2. **Raster Resizing**:
   - All rasters were resized to a standard 1 km resolution to ensure consistency across all spatial variables and facilitate the subsequent integration of the data layers.
3. **Vector Distribution and Support Maps**:
   - Risk maps were generated by combining vector distribution maps (**Figure S1**) with six supporting maps, which included elevation, mean annual temperature, precipitation, vector presence, population density, and median daily income (**Figure S2**). The sources of these data are detailed in Supplementary Table S1.
4. **Calculation of Risk Scale**:
   - The individual reclassified rasters were summed to produce a composite risk map, with a risk scale ranging from 0 to 14. This scale reflects the cumulative suitability for arboviral transmission, based on environmental and socioeconomic factors. However, the Culex spp. risk map had a slightly modified scale (0–12) due to the exclusion of nonhuman primate data, as there is limited evidence of WNV antibodies in nonhuman primates in Latin America.
   - **Exceptions**: In regions lacking data for median daily income (e.g., Venezuela and the Colombian Amazon), a mean value of 1 was assigned in the reclassification. Additionally, the risk map for Haemagogus spp. was limited by the scarcity of distribution data, which restricted the representation of Yellow Fever Virus (YFV) risk. Similarly, the lack of data regarding the environmental preferences of Sabethes spp. prevented the creation of a risk map for this vector.
5. **Data Sources**:
   - Data sources for the vector distribution maps and supporting environmental and socioeconomic variables are provided in Supplementary **Table S1**. These sources were carefully selected to ensure data accuracy and consistency throughout the analysis.
6. **Assumptions and Corrections**:
   - It is assumed that the ecological ranges provided in Table S2 accurately represent the species' preferences for temperature and precipitation, and that the population and income data are robust indicators of vulnerability to arboviral transmission in the study region.
   - The absence of data for certain regions (e.g., Venezuela and parts of the Colombian Amazon) was assumed to have minimal impact on the overall findings, given the assignment of mean values in these areas.
   - No major corrections were applied to the raw data. However, regions lacking daily income data were corrected by assigning a mean value in the reclassification process. The absence of specific data for *Haemagogus* spp. and *Sabethes* spp. was acknowledged and accounted for in the limitations of the risk maps.

Supplementary tables.

Table S1. Description of spatial variables. Variables used for the elaboration of risk maps, including corresponding value ranges, data type and source.

Variable	Ranges / Count	Description	Source
Elevation	0 – 5000 m	Data resolution: 1 arc second grid cell (30 m). Data divided into tiles extending over 1° x 1° lat/long in geographic projection	SRTM V3 digital elevation dataset (NASA JPL)[1]
Population density	0 – 1000 persons/km²	Data resolution: number of people per 30 arc-second grid cell (1km). Estimates based on national censuses and population registers, and adjusted to country totals from the 2015 Revision of the UN	Gridded Population of the World V4.11[2]

		World Population Prospects.<br>CC-BY-4.0	
Median daily income	1.5 – 25.5 US$ per capita	Welfare estimates by Global Administrative Unit Layers.<br>Data ranges from 2010 to 2018	Global Subnational Atlas of Poverty[3]
Precipitation	0 – 10923 mm	Data resolution: 30 arc seconds (1km). Annual trends derived from monthly temperature and precipitation.<br>CC-BY-SA-4.0	WorldClim BIO Variables V1–bio12[4]
Temperature	-10 – 32 °C		WorldClim BIO Variables V1–bio01[4]
Non-human primates	105017 cells	Occurrence data. Species distribution	IUCN Red List[5]
Vector:<br>*Aedes aegypti*<br>*Aedes albopictus*<br>*Culex* spp.<br>*Culicoides* spp.<br>*Haemagogus* spp.<br>*Sabethes* spp.	 5001 cells<br>3253 cells<br>1688 cells<br>273 cells<br>437 cells	Occurrence data<br>Sources: Global compendium of Ae. aegypti and Ae. albopictus occurrence[6]<br>GBIF[7] download DOIs:<br>Culex: https://doi.org/10.15468/dl.4xqee3<br>Culicoides: https://doi.org/10.15468/dl.3292ey<br>Haemagogus: https://doi.org/10.15468/dl.vgsez8<br>Sabethes: https://doi.org/10.15468/dl.ra3nn9	

Table S2. Reclassification of environmental variables. Variables range and assigned values correspond to the environmental preferences for each vector: 0=none or very low presence, 1=survival range, 2=optimum. MAP: Mean annual precipitation; MAT: Mean annual temperature. Sources of ecological ranges are indicated.

Vector	Variable	Range	Value	Vector	Variable	Range	Value	Vector	Variable	Range	Value	Vector	Variable	Range	Value	Vector	Variable	Range	Value
Aedes aegypti[8–11]	MAP (mm)	0-10	0	*Aedes albopictus*[8,10,12,13]	MAP (mm)	0-50	0	*Culex* spp.[14,15]	MAP (mm)	0-87	0	*Culicoides* spp.[16–18]	MAP (mm)	0-30	0	*Haemagogus* spp.[19–21]	MAP (mm)	0-100	0
		11-280	1			51-160	1			88-500	1			31-2010	1			100-1067	1
		281-800	2			161-1200	2			501-1676	2			2010-2590	2			1068-2762	2
		801-2200	1			1201-1700	1			1677-4000	1			2591-3000	1			2763-3800	1
		>2200	0			>1700	0			>4000	0			>3000	0			>3800	0
	MAT (C°)	-10-9	0		MAT (C°)	-10-5	0		MAT (C°)	-10-7	0		MAT (C°)	-10-8	0		MAT (C°)	-10-13	0
		10-19	1			6-17	1			8-13	1			9-11	1			14-19	1
		20-27	2			18-30	2			14-24	2			12-23	2			20-24	2
		28-34	1			31-34	1			25-30	1			24-27	1			25-30	1
		>34	0			>34	0			>30	0			>27	0			>30	0
	Elevation (m)	0-800	1		Elevation (m)	0-750	1		Elevation (m)	0-800	1		Elevation (m)	0-400	1		Elevation (m)	0-318	1
		801-1700	2			751-1800	2			801-2000	2			401-1830	2			319-784	2
		1701-2130	1			1801-3000	1			2001-2250	1			1830-3750	1			785-2300	1
		>2130	0			>3000	0			>2250	0			>3750	0			>2300	0

Table S3. Reclassification of vectors, hosts and socioeconomic variables. The occurrence of vectors and non-human primates was assigned a value of 1, or 0 in case of non-occurrence. Values assigned to median daily income range (US$) are based on World Bank poverty indicators[22]: 0=vulnerable population, 1=vulnerable population/middle income, 2=middle income population. Values assigned to population density (persons per km²) are based on the World Bank's urbanization indices for Latin America[23]: 0=low density, 1=moderate density, 2=high density.

**Variable**	**Range**	**Value**
Non-human primates (observation)[7]	missing	0
	present	2
Vector (observation)[6,7]	missing	0
	present	2
Population density (persons per sqkm)[2]	0-300	0
	301-500	1
	501-1000	2
Daily income (US$)[3]	20-25	0
	11-19	1
	0-10	2

Supplementary Figures

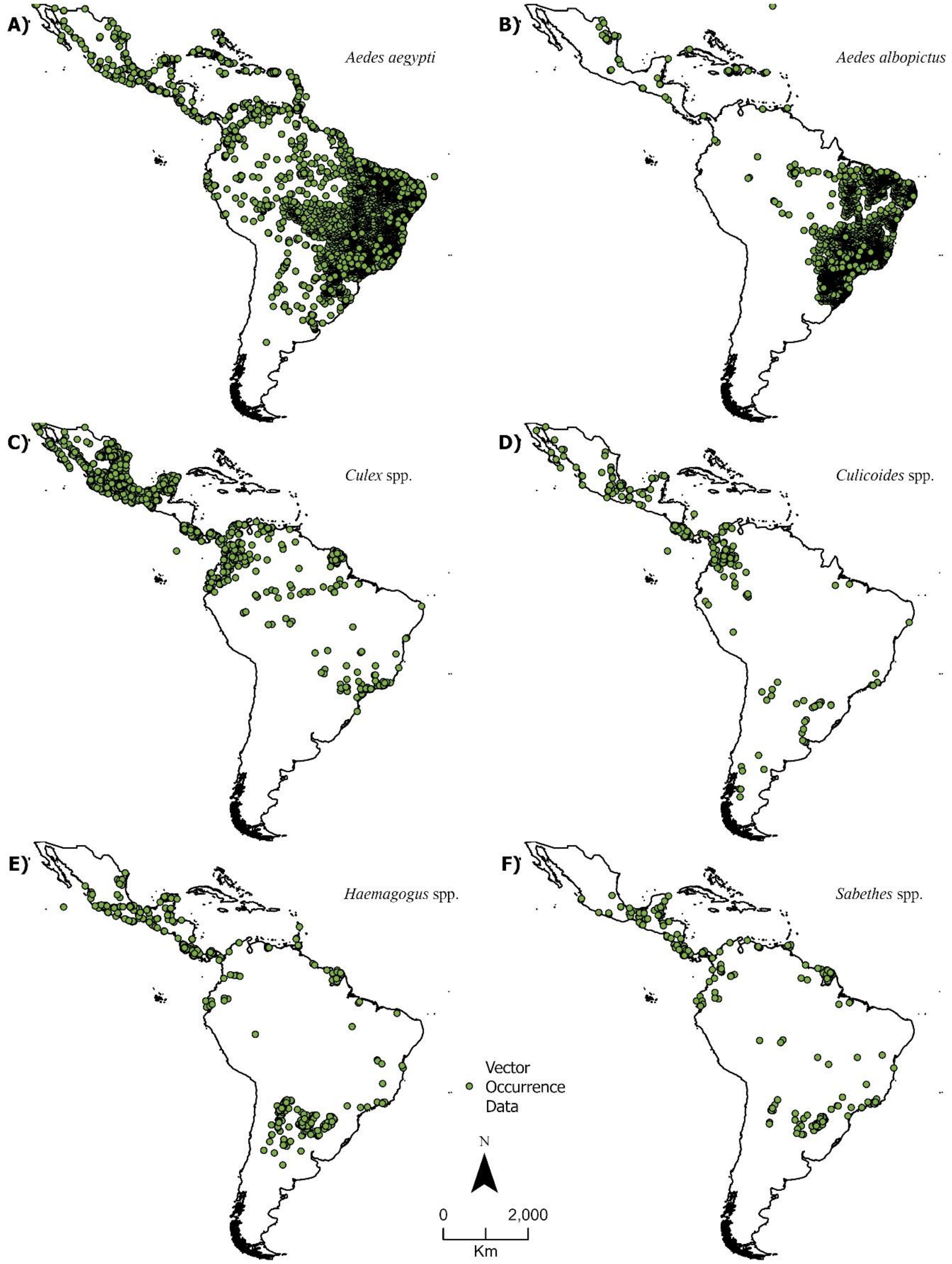

Figure S1. Vector distribution maps. A. *Aedes albopictus*; B. *Aedes Aegypti*; C. *Culex* spp.; D. *Culicoides* spp.; E. *Haemagogus* spp. F) *Sabethes* spp. Maps description and sources are included in Table S1 (appendix p 1). All maps were created with ArcGIS Pro 2.8[24].

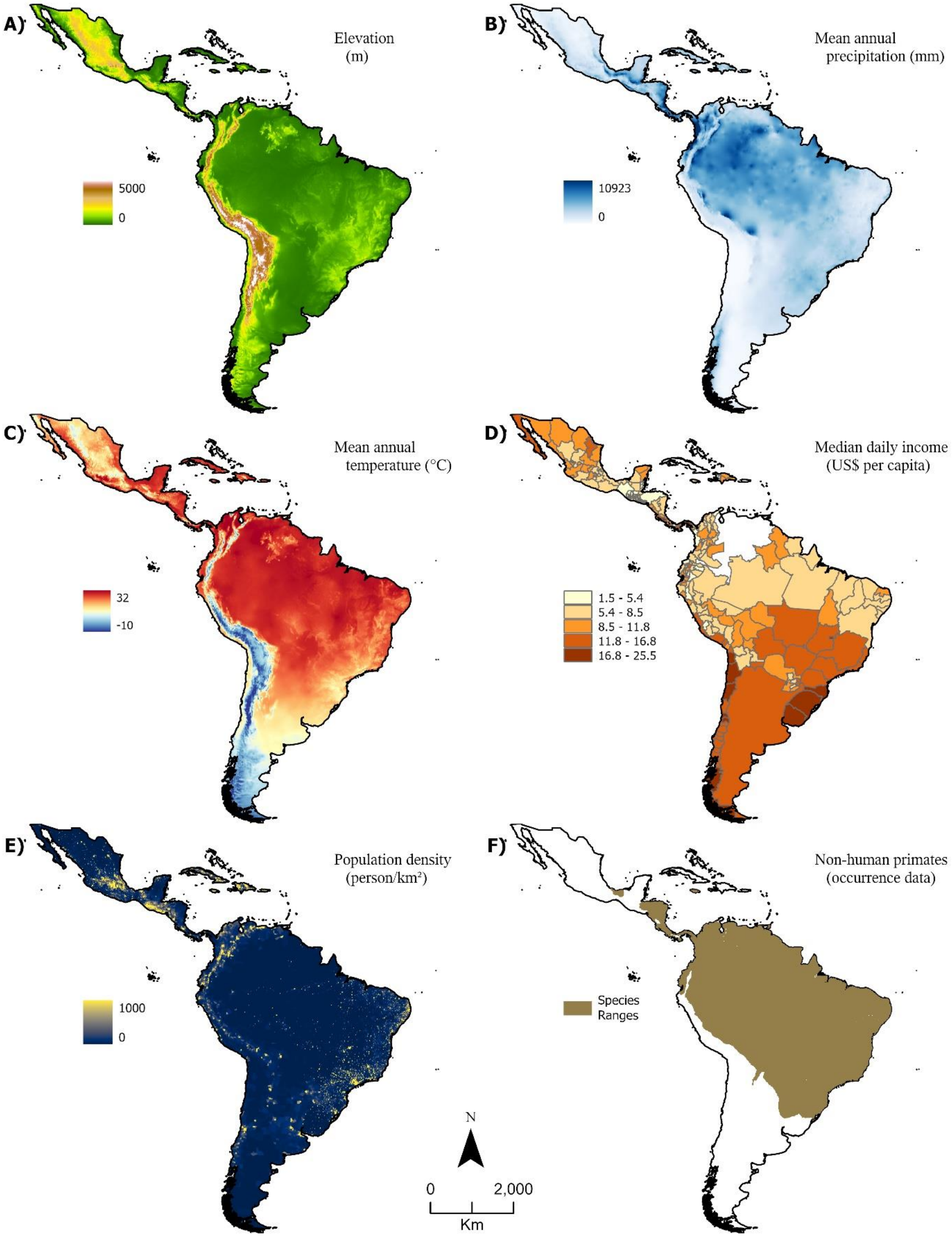

Figure S2. Support maps. A. Elevation (m); B. Mean annual precipitation (mm); C. Mean annual temperature (°C); D. Median daily income (US$ per capita); E. Population density (persons/km^2); Non-humane primates

(occurrence data). Maps description and sources are included in Table S1 (appendix p 1). All maps were created with ArcGIS Pro 2.8[24].

Supplementary references

1. Farr, T. G. *et al.* The Shuttle Radar Topography Mission. *Rev. Geophys.* **45**, RG2004 (2007).

2. Center for International Earth Science Information Network - CIESIN -. *Gridded Population of the World, Version 4 (GPWv4.11): Population Density Adjusted to Match 2015 Revision of UN WPP Country Totals, Revision 11*. (2018) doi:10.7927/H4F47M65.

3. World Health Organization. Introducing the second edition of the World Bank's Global Subnational Atlas of Poverty. https://blogs.worldbank.org/opendata/introducing-second-edition-world-banks-global-subnational-atlas-poverty (2021).

4. Hijmans, R. J., Cameron, S. E., Parra, J. L., Jones, P. G. & Jarvis, A. Very high resolution interpolated climate surfaces for global land areas. *Int. J. Climatol.* **25**, 1965–1978 (2005).

5. IUCN. The IUCN Red List of Threatened Species. Version 2022-2. https://www.iucnredlist.org (2022).

6. Kraemer, M. U. G. *et al.* The global compendium of Aedes aegypti and Ae. albopictus occurrence. *Sci. Data* **2**, 150035 (2015).

7. GBIF.org. Global Biodiversity Information Facility. https://www.gbif.org/ (2018).

8. Reinhold, J., Lazzari, C. & Lahondère, C. Effects of the Environmental Temperature on Aedes aegypti and Aedes albopictus Mosquitoes: A Review. *Insects* **9**, 158 (2018).

9. de Souza, S. J. P. *et al.* Spatial and Temporal Distribution of Aedes aegypti and Aedes albopictus Oviposition on the Coast of Paraná, Brazil, a Recent Area of Dengue Virus Transmission. *Trop. Med. Infect. Dis.* **7**, 246 (2022).

10. Laporta, G. Z. *et al.* Global Distribution of Aedes aegypti and Aedes albopictus in a Climate Change Scenario of Regional Rivalry. *Insects* **14**, 49 (2023).

11. Portilla Cabrera, C. V. & Selvaraj, J. J. Geographic shifts in the bioclimatic suitability for Aedes aegypti under climate change scenarios in Colombia. *Heliyon* **6**, e03101 (2020).

12. Cunze, S., Kochmann, J., Koch, L. K. & Klimpel, S. Aedes albopictus and Its Environmental Limits in Europe. *PLoS One* **11**, e0162116 (2016).

13. Echeverry-Cárdenas, E., López-Castañeda, C., Carvajal-Castro, J. D. & Aguirre-Obando, O. A. Potential geographic distribution of the tiger mosquito Aedes albopictus (Skuse, 1894) (Diptera: Culicidae) in current and future conditions for Colombia. *PLoS Negl. Trop. Dis.* **15**, e0008212 (2021).

14. Lorenz, C., de Azevedo, T. S. & Chiaravalloti-Neto, F. Impact of climate change on West Nile virus distribution in South America. *Trans. R. Soc. Trop. Med. Hyg.* **116**, 1043–1053 (2022).

15. Arora, A. K., Sim, C., Severson, D. W. & Kang, D. S. Random Forest Analysis of Impact of Abiotic Factors on Culex pipiens and Culex quinquefasciatus Occurrence. *Front. Ecol. Evol.* **9**, (2022).

16. Leta, S. *et al.* Modeling the global distribution of Culicoides imicola: an Ensemble approach. *Sci. Rep.* **9**, 14187 (2019).

17. Veggiani Aybar, C. A., Díaz Gomez, R. A., Dantur Juri, M. J., Lizarralde de Grosso, M. S. & Spinelli, G. R. Potential Distribution Map of Culicoides insignis (Diptera: Ceratopogonidae), Vector of Bluetongue Virus, in Northwestern Argentina. *J. Insect Sci.* **16**, (2016).

18. Mukhopadhyay, E., Hazra, S., Saha, G. K. & Banerjee, D. Altitudinal variation and bio-climatic variables influencing the potential distribution of Culicoides orientalis Macfie, 1932, suspected vector of Bluetongue virus across the North Eastern Himalayan belt of Sikkim. *Acta Trop.* **176**, 402–411 (2017).

19. Celone, M. *et al.* An ecological niche model to predict the geographic distribution of Haemagogus janthinomys, Dyar, 1921 a yellow fever and Mayaro virus vector, in South America. *PLoS Negl. Trop. Dis.* **16**, e0010564 (2022).

20. Couto-Lima, D. *et al.* Seasonal population dynamics of the primary yellow fever vector Haemagogus leucocelaenus (Dyar & Shannon) (Diptera: Culicidae) is mainly influenced by temperature in the Atlantic Forest, southeast Brazil. *Mem. Inst. Oswaldo Cruz* **115**, (2020).

21. Hamrick, P. N. *et al.* Geographic patterns and environmental factors associated with human yellow fever presence in the Americas. *PLoS Negl. Trop. Dis.* **11**, e0005897 (2017).

22. World Bank. LAC Equity Lab. (2020).

23. Buys, P., Chomitz, K. M. & Thomas, T. S. *Quantifying The Rural-Urban Gradient In Latin America And The Caribbean*. (The World Bank, 2005). doi:10.1596/1813-9450-3634.

24. Esri inc. ArcGis Pro. (2021).
