# Supplementary material for: Transfusion-transmitted infections: risks and mitigation strategies for Oropouche virus and other emerging arboviruses in Latin America and the Caribbean
Source: Lancet Reg Health Am. 2025 May 5;46:101089. doi: 10.1016/j.lana.2025.101089 (PMC12127558; doi:10.1016/j.lana.2025.101089)
Supplement: Translated Summary [file mmc2.pdf]

__Editorial Disclaimer:__ This translation in Spanish was submitted by the authors and we reproduce it as supplied. It has not been peer-reviewed. Our editorial processes have only been applied to the original abstract in English, which should serve as a reference for this manuscript.

Resumen

Los arbovirus representan una carga significativa para la salud pública en América Latina y el Caribe debido a infecciones generalizadas y potencialmente graves, como la microcefalia y la artralgia. Más allá de la transmisión vectorial canónica, la magnitud y los factores de riesgo de las infecciones transmitidas por transfusión (TTIs por sus siglas en inglés) no están claros. En este reporte narrativo utilizamos análisis de datos virológicos como sintomatología de la infección, períodos de viremia y cargas virales, para argumentar que los virus del dengue, Oropouche, Zika, fiebre amarilla y Chikungunya representan un riesgo poco estudiado de TTIs. Un análisis de datos socioeconómicos mostró que las tasas de donación de sangre se correlacionaron con el producto interno bruto ($r=0,53$, $p=0,0021$) y el gasto en salud ($r=0,5$, $p=0,0045$), lo que resalta que las limitaciones de recursos afectan el tamizaje sanguíneo. Los mapas de riesgo basados en la presencia de vectores y variables ecológicas indicaron que América Central y la costa noroeste de Brasil son zonas de alto riesgo, lo que hace que la vigilancia, el control de vectores, la vacunación y el tamizaje sanguíneo rentable sean esenciales para mitigar las TTIs, incluidas las causadas por los virus Zika y, potencialmente, Oropouche en mujeres embarazadas.
