# Supplementary material for: Transfusion-transmitted infections: risks and mitigation strategies for Oropouche virus and other emerging arboviruses in Latin America and the Caribbean
Source: Lancet Reg Health Am. 2025 May 5;46:101089. doi: 10.1016/j.lana.2025.101089 (PMC12127558; doi:10.1016/j.lana.2025.101089)
Supplement: Translated Summary [file mmc3.pdf]

Editorial Disclaimer: This translation in Portuguese was submitted by the authors and we reproduce it as supplied. It has not been peer-reviewed. Our editorial processes have only been applied to the original abstract in English, which should serve as a reference for this manuscript.

Resumo

Os arbovírus representam uma carga significativa para a saúde pública na América Latina e no Caribe devido a infecções generalizadas e potencialmente graves, como microcefalia e artralgia. Além da transmissão vetorial canônica, a magnitude e os fatores de risco das infecções transmitidas por transfusão (TTIs, na sigla em inglês) não estão claros. Neste relatório narrativo, utilizamos análises de dados virológicos, como sintomatologia da infecção, períodos de viremia e cargas virais, para argumentar que os vírus da dengue, Oropouche, Zika, febre amarela e Chikungunya representam um risco pouco estudado de TTIs. Uma análise de dados socioeconômicos mostrou que as taxas de doação de sangue se correlacionaram com o produto interno bruto (r=0,53, p=0,0021) e os gastos com saúde (r=0,5, p=0,0045), destacando que as limitações de recursos afetam a triagem sanguínea. Mapas de risco baseados na presença de vetores e variáveis ecológicas indicaram que a América Central e a costa noroeste do Brasil são zonas de alto risco, tornando essenciais a vigilância, o controle de vetores, a vacinação e a triagem sanguínea econômica para mitigar as TTIs, incluindo aquelas causadas pelos vírus Zika e, potencialmente, Oropouche em mulheres grávidas.
